# Auswirkungen der Pflegevisite auf die Prozessverantwortliche Pflege: Eine Evaluationsstudie auf der Intensivstation

**DOI:** 10.1007/s00063-024-01163-7

**Published:** 2024-06-25

**Authors:** Dirk Johnen, Lars Krüger, Thomas Mannebach, Francesco Squiccimarro, Gero Langer, Elke Hotze

**Affiliations:** 1 Intensivstation E 0.1, Herz- und Diabeteszentrum NRW, Universitätsklinikum der Ruhr-Universität Bochum, Georgstraße 11, 32345 Bad Oeynhausen, Deutschland; 2 Stabsstelle Projekt- und Wissensmanagement/Pflegeentwicklung Intensivpflege, Pflegedirektion, Herz- und Diabeteszentrum NRW, Universitätsklinikum der Ruhr-Universität Bochum, Georgstraße 11, 32545 Bad Oeynhausen, Deutschland; 3 Intensivstation A 1.4, Herz- und Diabeteszentrum NRW, Universitätsklinikum der Ruhr-Universität Bochum, Georgstraße 11, 32345 Bad Oeynhausen, Deutschland; 4 Institut für Gesundheits- und Pflegewissenschaft, German Center for Evidence-based Nursing, Medizinische Fakultät, Martin-Luther-Universität Halle-Wittenberg, Magdeburger Straße 8, 06112 Halle (Saale), Deutschland; 5 Fakultät für Wirtschafts- und Sozialwissenschaften, Hochschule Osnabrück, Caprivistraße 30a, 49076 Osnabrück, Deutschland

**Keywords:** Beratung, Patientenzentrierung, Pflegeplanung, Pflegeprozess, Krankenhaus, Counseling, Hospitals, Nursing process, Patient care planning, Patient-centered care

## Abstract

**Hintergrund:**

An einem Universitätsklinikum wurde die Pflegevisite (PV) auf einer Intensivstation (ITS) nach einer Pilotphase im Jahr 2017 implementiert. Bisher fehlt es an publizierten Erkenntnissen zur Auswirkung der PV auf das Pflegeorganisationssystem Primary Nursing (Prozessverantwortliche Pflege [PP]).

**Ziel:**

Primäres Ziel war, Auswirkungen der PV auf die PP aus Sicht der Pflegenden zu untersuchen. Als sekundäre Ziele sollten mit einem Vergleich zu den Ergebnissen der Pilotierungsphase (t_0_) u. a. weitere Auswirkungen, Rahmenbedingungen der PV und die Gesamtbewertung ermittelt werden.

**Methode:**

Die quantitative Evaluationsstudie erfolgte mittels eines standardisierten Fragebogens.

**Ergebnisse:**

Die Befragung fand von 09. bis 10.2023 (t_1_) mit einem Rücklauf von 74,6 % (*n* = 47) statt. Auf einer Skala von 1–6 (stimme voll zu; stimme überhaupt nicht zu) trug die PV bei 100,0 % der prozessverantwortlich Pflegenden (PP; *n* = 8) und 77,0 % der Pflegenden ohne Prozessverantwortung (P; *n* = 30) mit den Stufen 1–3 (*p* = 0,328) dazu bei, die Pflegeplanung bei prozessverantwortlich betreuten Patientinnen und Patienten zu evaluieren. Die PV stellte eine Unterstützung für die Umsetzung der PP dar (PP: 100,0 %, *n* = 8; P: 79,5 %, *n* = 31; *p* = 0,318) und hatte einen statistisch signifikanten Effekt (r = 0,97; *p* = 0,035) auf die Verbesserung der Pflegequalität und Pflegeplanung für prozessverantwortlich betreute Patientinnen und Patienten. Die Teilnehmenden gaben mit den Stufen 1–3 an, dass die Patientinnen und Patienten durch die PV bewusster in den Fokus der Pflege gerückt werden (t_1_: 74,4 %, *n* = 35; t_0_: 86,4 %, *n* = 38; *p* = 0,953). Die PV sollte wöchentlich stattfinden und wurde im Median mit einer 2 (IQR t_1_: 1–3; t_0_:1–2) bewertet.

**Schlussfolgerung:**

PV unterstützen die Umsetzung von PP sowie die Patientinnen- und Patientenzentrierung auf der ITS.

**Zusatzmaterial online:**

Zusätzliche Informationen sind in der Online-Version dieses Artikels (10.1007/s00063-024-01163-7) enthalten.

## Abstract (en)

**Background:**

After a pilot phase in 2017, nursing visits (PV) were implemented in an intensive care unit (ICU) at a university hospital. So far, published findings on the impact of PV on the primary nursing organisation system (process-responsible nursing [PP]) could not be identified.

**Aim:**

Primary aim was to investigate the effects of PV on PP from the nurses perspective. Secondary aims included comparison with the results of the pilot phase (t_0_) to determine further effects, general conditions of the PP and the overall evaluation.

**Methods:**

A quantitative evaluation study using a standardised questionnaire was used.

**Results:**

The survey was conducted in September to October 2023 (t_1_) with a response rate of 74.6% (*n* = 47). On a scale of 1–6 (strongly agree; strongly disagree), 100.0% of the process-responsible nurses (PP; *n* = 8) and 77.0% of the nurses without process responsibility (P; *n* = 30) rated the PV at levels 1–3 (*p* = 0.328) as contributing to the evaluation of care planning for patients with process responsibility. PV provided support for the implementation of PP (PP: 100.0%, *n* = 8; P: 79.5%, *n* = 31; *p* = 0.318) and had a statistically significant effect (r = 0.97, *p* = 0.035) on improving the quality of care and care planning for patients with procedural responsibility. The nurses indicated with levels 1–3 that the patients were more consciously brought into the focus of nursing care through the PV (t_1_: 74.4%, *n* = 35; t0: 86.4%, *n* = 38; *p* = 0.953). The PV should take place weekly and was rated with a median of 2 (IQR t_1_: 1–3; t_0_:1–2).

**Conclusion:**

PV support the implementation of PP and patient-centred care in the ICU.

## Einleitung

Eine erste Beschäftigung mit der Thematik Pflegevisite (PV) fand in deutschen Krankenhäusern bereits in den 1980er-Jahren statt [1]. Später erfolgte auch eine Übertragung des Konzepts auf die Intensivstationen (ITS). Görres et al. [2] berichten, dass rund ein Drittel der Kliniken in 5 Bundesländern eine PV durchführen. Umfassende Forschungsprojekte zu den Auswirkungen der PV auf die pflegerische Versorgung in Deutschland blieben bisher aus. Die nachfolgende Evaluationsstudie fokussiert den Einsatz der PV im Zusammenhang mit *Prozessverantwortlicher Pflege* (PP) auf der ITS.

### Hintergrund

Eine PV wird je nachdem, welche Zwecke und Ziele verfolgt werden, unterschiedlich definiert. Dazu gehört z. B. die Form des Führungs- und Supervisionsinstruments oder ein kooperativer Entscheidungsprozess, bei dem die Beteiligung der Patientinnen und Patienten in den Mittelpunkt gestellt wird [3]. Der Einsatz der PV ist nicht an einen bestimmten Fachbereich gekoppelt. So findet sich eine Umsetzung neben z. B. dem palliativmedizinischen Setting [4], der Onkologie [5], der Anästhesie [6] v. a. auch auf der ITS [7–9]. Losgelöst von der PV ist grundsätzlich die interdisziplinäre Visite zu betrachten, die täglich stattfinden sollte [10].

PV finden im internationalen Kontext der Intensivpflege zum Teil mehrfach täglich [11, 12] bis einmal [13] oder mehrmals wöchentlich [7] mit unterschiedlichen Teilnehmenden (TN) statt. Neben der Patientinnen- und Patientenzentrierung [14] haben PV u. a. Medikationsfehler [15], Reduktion von Infektionen [16] und Dekubitus [17], hämodynamisches Monitoring [18], die Begleitung von Eltern bzw. Angehörigen [12], aber auch die Planung der pflegerischen Versorgung [11] im Fokus.

Im Herz- und Diabeteszentrum NRW (HDZ NRW), Universitätsklinikum der Ruhr-Universität Bochum, wird die PV nach einer erfolgreichen Pilotierungsphase auf einer Pilot-ITS seit 2017 umgesetzt [19] und wurde über die Jahre auf weitere ITS ausgeweitet. Im Jahr 2022 erfolgte auf derselben Pilot-ITS zusätzlich die Einführung und Implementierung von PP [20]. Das Pflegesystem ist mit dem international bekannten *Primary Nursing* vergleichbar (PN; [21]). Hierbei hat eine Pflegefachperson die Hauptverantwortung für den Pflegeprozess inkl. Erstellung der Pflegeplanung, Evaluation der erreichten Pflegeziele und Anpassung der geplanten Pflegemaßnahmen [21].

Die PV kann unter dieser Prämisse ein Instrument sein, um die Pflegequalität, pflegerische Kontinuität sowie die Informations- und Wissensweitergabe zu gewährleisten und somit Teilaspekten der genannten Elemente des PN begegnen zu können [8]. Bisher fehlt es an Forschungsprojekten, die die PV im Kontext von PP bzw. PN fokussieren.

### Ziele

**Abb. 1:**
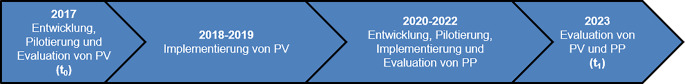
Zeitstrahl der Entwicklungen und Evaluationen von Pflegevisiten (*PV*) und Prozessverantwortlicher Pflege (*PP*)

Primäres Ziel dieser Evaluationsstudie war es, die Auswirkungen der PV auf PP aus Sicht der Pflegenden zu untersuchen. Als sekundäre Ziele sollten mit einem Vergleich zu den Ergebnissen der Pilotierungsphase weitere Auswirkungen und Rahmenbedingungen der PV, die Gesamtbewertung sowie die Anzahl und Dauer der PV im Jahr 2023 ermittelt werden (Abb. 1).

## Methodik

### Studiendesign

Nach erfolgter Pilotierungsphase der PV im Jahr 2017 wurde im Jahr 2023 eine Evaluationsstudie (t_1_) in einem nichtexperimentellen Forschungsdesign durchgeführt (Abb. 1)*. *Die Berichterstattung folgt dem Strengthening-the-reporting-of-observational-studies-in-epidemiology(STROBE)-Statement [22].

### Ethische Überlegungen

Eine Stellungnahme der zuständigen Ethikkommission wurde zunächst nicht eingeholt, da Pflegende als Zielgruppe, wie auch in t_0_ [19], nicht als vulnerabel eingeschätzt wurden. Der zuständige Betriebsrat stimmte der Datenerhebung zu. Die Ethikkommission der Medizinischen Fakultät der Ruhr-Universität Bochum mit Sitz in Bad Oeynhausen bewertete die Evaluationsstudie im Nachgang an die Datenaufnahme als ethisch unbedenklich (Aktenzeichen 2024-1200).

### Setting und Interventionen

Das HDZ NRW verfügt über insgesamt 6 ITS mit 104 Intensivbetten. Die Pilot-ITS hat 23 Planbetten mit dem medizinischen Schwerpunkt Thorax- und Kardiovaskularchirurgie.

#### Pflegevisite

Für die wöchentlich stattfindende PV ist ein aus 4 Personen bestehendes pflegerisches Visitationsteam verantwortlich, von denen jeweils mindestens eine Person für die PV freigestellt wird. Alle Pflegenden im Visitationsteam verfügen über jeweils mehr als 15 Jahre Berufserfahrung im Fachbereich und schlossen unterschiedliche weitere Qualifikationen ab. Dazu gehören die Weiterbildungen Intensivpflege und Anästhesie (FWB), Palliative Care, Praxisanleitung oder ein pflegebezogenes Studium. Die PV wird in einer kollegial-beratenden Form umgesetzt.

Patientinnen und Patienten werden für die PV ausgewählt, wenn:

- diese prozessverantwortlich pflegerisch versorgt werden (≥ 3 Tage Aufenthalt auf der ITS) oder
- Bedarf seitens der Pflegenden, Patientinnen und Patienten oder Angehörigen angemeldet wird.

Am Visitationstag stimmt die visitierende Pflegefachperson einen Termin mit den zuständigen prozessverantwortlich Pflegenden (PP) oder Pflegenden ohne Prozessverantwortung (P) ab. Zum vereinbarten Zeitpunkt stellt die zuständige PP oder P der visitierenden Pflegefachperson die Patientin oder den Patienten vor. Sofern möglich werden diese aktiv mit in den PV-Prozess eingebunden. Anschließend erfolgt die Benennung der identifizierten pflegerischen Probleme, Ziele und Maßnahmen. Im Rahmen einer kollegialen Beratung werden die einzelnen Punkte eruiert und ggf. Anpassungen vorgenommen. Die Ergebnisdokumentation der PV erfolgt digital im Patient-data-management-System (PDMS). Abschließend wird ein gegenseitiges Feedback gegeben [19].

#### Prozessverantwortliche Pflege

PP als Pflegeorganisationsmodell ist an PN angelehnt und stellt ein flexibles System dar, das die bestmögliche pflegerische Versorgung von Patientinnen und Patienten im Rahmen der verfügbaren Ressourcen innerhalb einer Organisationseinheit ermöglichen soll [21]. PP auf der Pilot-ITS besteht aus den 4 Kernelementen von PN [21, 23]:

- Übertragung der Verantwortung des Pflegeprozesses auf eine feste Pflegefachperson,
- Kontinuität,
- direkte Kommunikation,
- Übernahme der Verantwortung für die Pflegequalität der zugeordneten Patientinnen und Patienten über die gesamte Aufenthaltsdauer auf der ITS.

Das Konzept im HDZ NRW sieht einen Einschluss aller Patientinnen und Patienten ab dem 3. Tag auf der ITS vor. Eine PP ist für maximal 2 Patientinnen und Patienten zuständig und erstellt u. a. eine umfassende Sozialanamnese sowie eine schriftliche Pflegeplanung [20]. Beides wird in der PV aktiv gesichtet und miteingebunden. In der Abwesenheit der PP stellen P die weitere Versorgung gemäß der Pflegeplanung sicher. Sie nehmen ebenso stellvertretend an der PV teil, falls die PP nicht im Tagdienst vor Ort ist.

### Stichprobe

Im Jahr 2023 waren auf der Pilot-ITS insgesamt 98 Pflegende mit einer mindestens 3‑jährigen fachschulischen Pflegeausbildung beschäftigt, von denen 42,9 % (*n* = 42) über 10 Jahre Berufserfahrung aufwiesen. Überdies verfügten viele Pflegende über einen Bachelorabschluss in der Pflege oder eine abgeschlossene Weiterbildungen wie die FWB, Praxisanleitung oder andere Weiterbildungen. Insgesamt 10 Pflegende (10,2 %) hatten die Rolle PP. Im Zeitraum der Datenaufnahme waren 63 Pflegende aktiv anwesend und wurden zur Teilnahme an der Studie eingeladen.

### Messmethode und Datenerhebung

Zur Datenerhebung wurde ein eigens erstellter Fragebogen eingesetzt. Der Entwicklungs- und Überarbeitungsprozess fand in 3 Phasen statt: Konzeptionsphase, Konstruktionsphase und Testphase [24].

Als Grundlage diente der in t_0_ erstellte und mit einem standardisierten Pretest geprüfte Fragebogen mit 17 Items [19]. Es erfolgte eine Erweiterung um 7 Items, die spezifisch in Bezug auf PP (3 Items), zu den Kompetenzen der visitierenden Personen (1 Item) sowie zur Soziodemografie (3 Items) erstellt wurden.

Zum Einsatz kamen geschlossene, halboffene und offene Fragen. Diese wurden durch 6‑stufige Likert-Skalen, dichotome Antwortmöglichkeiten sowie durch die Ergänzung von offenen Antwortoptionen abgebildet. Nach einem erneuten standardisierten Pretest mit 6 Pflegefachpersonen, die die Zielgruppe abbildeten, erfolgten kleinere sprachliche Korrekturen bei den neu hinzugefügten Items. Der gesamte Prozess wurde von einer Professorin für Pflegewissenschaft begleitet. Der Fragebogen wird auf Anfrage beim korrespondierenden Autor zur Verfügung gestellt.

Alle im Erhebungszeitraum anwesenden Pflegenden auf der Pilot-ITS wurden in einer Abteilungsbesprechung, in den Dienstübergaben sowie per E‑Mail zur freiwilligen und anonymen Teilnahme an dieser Studie eingeladen und zusätzlich schriftlich aufgeklärt.

Es erfolgte eine postalische Versendung des Fragebogens. Zur anonymen Teilnahme stand eine Sammelbox bereit. Die Rücklaufzeit betrug 4 Wochen (04.09.2023 bis 01.10.2023 [t_1_]). Zur Steigerung des Rücklaufs erfolgte neben kontinuierlichen Ansprachen in der Übergabezeit im Plenum nach 2 Wochen eine erneute Erinnerung via E‑Mail.

Die Anzahl und Dauer der PV im Jahr 2023 wurde aus dem PDMS ausgelesen.

### Statistische Methoden

Der eingesetzte Fragebogen bestand aus nominalen und metrischen Skalen, die quantifiziert und mit R (R Version 4.3.3, Wien, Österreich) analysiert wurden. Zur Prüfung auf Normalverteilung der Daten wurde der *Shapiro-Wilk-Test* eingesetzt. Die Berichterstattung erfolgte entsprechend mit Mittelwert und Standardabweichung (SD; bei Normalverteilung) oder Median und Interquartilsabstand (IQR; bei nichtvorhandener Normalverteilung). Fehlende Daten wurden ausgewiesen. In t_1_ wurden isolierte Fragen aufgrund zum Teil kleiner Teilnehmendenzahlen mithilfe des *exakten Fisher-Tests* auf Unabhängigkeit in Gruppen von PP und P getestet. Ergebnisse mit *p*-Werten > 0,05 konnten keine statistische Signifikanz hervorheben, da die befragten PP und P sich in ihren Aussagen nicht deutlich unterscheiden. Das Studiendesign und der zeitliche Abstand der Datenerhebung bot den geeigneten Hintergrund, um den Einfluss der PV auf die PP zu ermitteln. Durch die bereits gewonnenen Daten in t_0_ wurden, je nach Datenverteilung, einzelne klinisch relevante Fragen bzw. Variablen einer Korrelationsprüfung mit *Pearsons Korrelationstest* unterzogen.

## Ergebnisse

Der Rücklauf betrug 74,6 % (*n* = 47), von denen 17,0 % (*n* = 8) PP waren. 42,6 % (*n* = 20) der TN verfügten über die 3‑jährige fachschulische Pflegeausbildung und/oder eine FWB (42,6 %, *n* = 20). 38,3 %, (*n* = 18) der TN hatten eine Berufserfahrung von über 10 Jahren (*ESM 1*).

Alle TN (100 %, *n* = 47) gaben an, dass die PV durchgeführt wird. Sie erhielten die Informationen dazu mehrheitlich durch Pflegende (63,8 %, *n* = 30; Tab. 1*)*.

**Tab. 1:** Informationsverbreitung zur Pflegevisite

Woher hast du die Information zur Pflegevisite erhalten? (Mehrfachnennung möglich, *n* = 47)								
	Stationsbesprechung	Nachschulung	Information via E‑Mail	Aushang	Pflegende	Praktische Durchführung der Pflegevisite	Einarbeitung	Sonstiges
Gesamt t_1_, *n*	28	8	15	10	30	29	10	1
Gesamt t_1_, %	59,6	17,0	31,9	21,3	63,8	61,7	21,3	2,1

Insgesamt 80,9 % (*n* = 38, Mehrfachantworten möglich) der TN nahmen mindestens einmal an einer PV bei PP-Patientinnen und Patienten teil; 70,2 % (*n* = 33) zusätzlich oder ausschließlich bei Patientinnen und Patienten, die nicht im PP-Programm waren. Zwei Personen (4,3 %) nahmen bisher nicht aktiv an einer PV teil.

### Auswirkungen der PV auf die PP

**Abb. 2:**
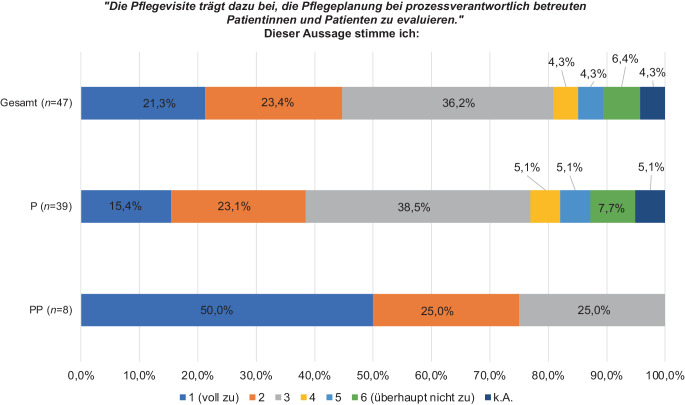
Auswirkung der Pflegevisite auf die Evaluation der Pflegeplanung bei prozessverantwortlich betreuten Patientinnen und Patienten

Auf einer Skala von 1–6 (stimme voll zu; stimme überhaupt nicht zu) trafen auf die Frage, ob die PV dazu beiträgt, die Pflegeplanung bei prozessverantwortlich betreuten Patientinnen und Patienten zu evaluieren, 100,0 % der PP (*n* = 8) und 77,0 % der P (*n* = 30) eine Auswahl bei den Stufen 1–3 (*p* = 0,328; Abb. 2*).*

**Abb. 3:**
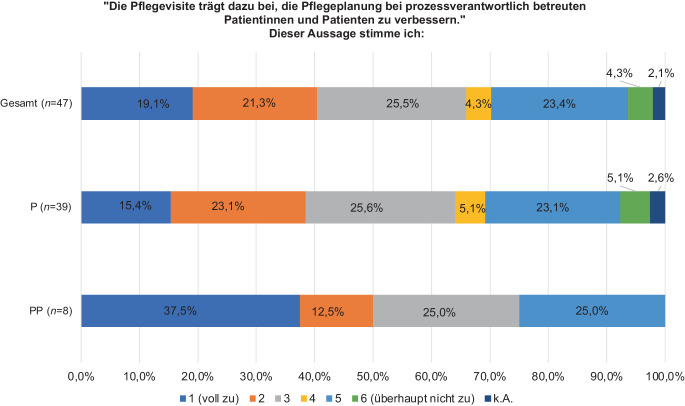
Auswirkung der Pflegevisite auf die Verbesserung der Pflegeplanung bei prozessverantwortlich betreuten Patientinnen und Patienten

Eine heterogenere Verteilung stellte sich auf derselben Skala in den Stufen 1–3 bei der Frage dar, ob die PV dazu beiträgt, die Pflegeplanung bei prozessverantwortlich betreuten Patientinnen und Patienten zu verbessern (PP: 75,0 %, *n* = 6; P: 64,1 %, *n* = 25; *p* = 0,624; Abb. 3).

**Abb. 4:**
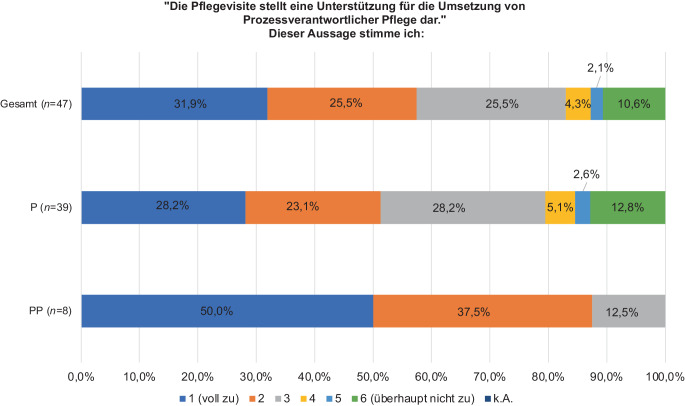
Auswirkung der Pflegevisite auf die Umsetzung von Prozessverantwortlicher Pflege

Es wurde mit den Stufen 1–3 mehrheitlich zugestimmt, dass die PV eine Unterstützung für die Umsetzung von PP darstellt (PP: 100,0 %, *n* = 8; P: 79,5 %, *n* = 31; *p* = 0,318; Abb. 4)*.*

### Auswirkungen der PV auf die Patientinnen und Patienten in t_1_ und t_0_

**Abb. 5:**
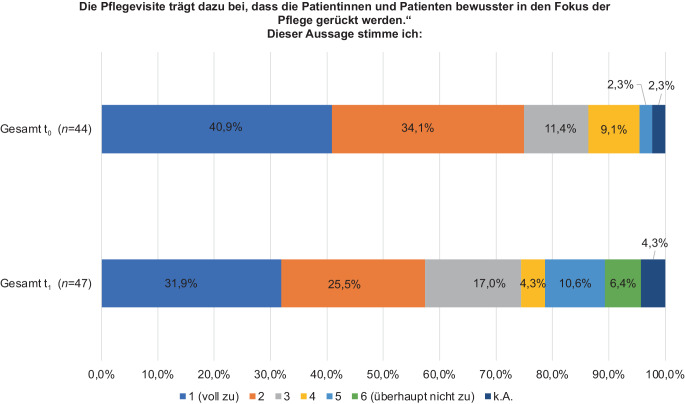
Auswirkung der Pflegevisite auf die Patientinnen- und Patientenzentrierung nach (t_1_) und vor Implementierung (t_0_) von Prozessverantwortlicher Pflege

Die Auswirkungen der PV auf die Patientinnen und Patienten wurden in t_1_ und t_0_ unter Einsatz einer 6‑stufigen Skala erhoben. Zur Patientinnen- und Patientenzentrierung erfolgte die Frage, ob die PV dazu beiträgt, dass die Patientinnen und Patienten bewusster in den Fokus der Pflege gerückt werden (1: stimme voll zu; 6: stimme überhaupt nicht zu). Hier stimmten in beiden Erhebungen die TN mehrheitlich (t_1_: 74,4 %, *n* = 35; t_0_: 86,4 %, *n* = 38) mit den Stufen 1–3 zu (*p* = 0,953; Abb. 5).

Die Auswirkungen der PV auf eine bewusstere Anpassung von pflegerischen Maßnahmen auf die Bedürfnisse der Patientinnen und Patienten wurden in t_1_ (70,2 %, *n* = 33) und t_0_ (70,5 %, *n* = 31) positiv mit den Stufen 1–3 bewertet (*p* = 0,868; Tab. 2)*.*

**Tab. 2:** Auswirkungen der Pflegevisite auf die Anpassung von pflegerischen Maßnahmen nach (t_1_) und vor Implementierung (t_0_) von Prozessverantwortlicher Pflege

„Mir hat die Pflegevisite geholfen, pflegerische Maßnahme bewusster an die Bedürfnisse der Patientinnen und Patienten anzupassen.“ Dieser Aussage stimme ich:							
	1 – stimme voll zu	2	3	4	5	6 – stimme überhaupt nicht zu	k. A.
Gesamt t_1_, *n*	14	8	11	4	7	2	1
Gesamt t_1_, % (*n* = 47)	29,8	17,0	23,4	8,5	14,9	4,3	2,1
Gesamt t_0_ *n*	9	16	6	3	5	1	4
Gesamt t_0_, % (*n* = 44)	20,5	36,4	13,6	6,8	11,4	2,3	9,1

Ähnlich verteilten sich die Angaben zu den Auswirkungen der PV auf die Pflegequalität (t_1_: 72,4 %, *n* = 34; t_0_: 70,4 %, *n* = 31; *p* = 0,837; Tab. 3). Eine verbesserte Pflegequalität wurde vor allem an einer patientinnen- und patientenorientierteren Pflege (t_1_: 78,7 %, *n* = 37; t_0_ 68,2 %, *n* = 30; Mehrfachnennungen möglich) festgemacht *(ESM 2).*

**Tab. 3:** Auswirkungen der Pflegevisite auf die Pflegequalität nach (t_1_) und vor Implementierung (t_0_) von Prozessverantwortlicher Pflege

„Die Pflegevisite bewirkt eine positive Veränderung der Pflegequalität.“ Dieser Aussage stimme ich:							
	1 – stimme voll zu	2	3	4	5	6 – stimme überhaupt nicht zu	k. A.
Gesamt t_1_, *n*	14	10	10	5	4	3	1
Gesamt t_1_, % (*n* = 47)	29,8	21,3	21,3	10,6	8,5	6,4	2,1
Gesamt t_0_, *n*	11	10	10	5	4	1	3
Gesamt t_0_, % (*n* = 44)	25,0	22,7	22,7	11,4	9,1	2,3	6,8

Die PV hatte nach Pearsons Korrelationstest einen statistisch signifikanten Effekt (r = 0,97, *p* = 0,035 für α < 0,05) auf die Verbesserung der Pflegequalität und Pflegeplanung für prozessverantwortlich betreute Patientinnen und Patienten.

### Rahmenbedingungen zur PV in t_1_ und t_0_

Die präferierte Durchführung der PV wurde in beiden Gruppen mehrheitlich mit einmal wöchentlich angegeben (t_1_: 70,2 %, *n* = 33; t_0_: 75,0 %, *n* = 33; *ESM 2*)*.*

Für die PV sollten in t_1_ und t_0_ vor allem Patientinnen und Patienten ausgewählt werden, die länger als 3 Tage auf der ITS verweilen (t_1_: 97,9 %, *n* = 46; t_0_: 88,6 %, *n* = 39). In t_1_ gaben zusätzlich 17,2 % (*n* = 8) der TN PP-Patientinnen und Patienten als Zielgruppe an, von denen 6 TN (75,0 %) selbst keine PP waren. Eine PV für alle Patientinnen und Patienten wurde ausschließlich in t_0_ mit 4,6 % (*n* = 2) gewünscht. Spezifische Krankheitsbilder (t_1_: 2,1 %, *n* = 1; t_0_: 13,5 %, *n* = 6) wurden als Einschlussgrund ebenfalls genannt.

Zur Dauer einer PV gaben die TN in t_1_ im Median 22,5 Min (IQR: 20–30) und in t_0_ 30 Min (IQR 20–30) an. Insgesamt wurden in t_1_ im Median eine Person (IQR 1–[1–2]) und in t_0_ 2 Personen (IQR: [1–2]–2) als Anzahl an visitierenden Personen angegeben.

In t_1_ erfolgte ergänzend die Frage nach den präferierten Qualifikationen der visitierenden Personen im Rahmen der PV. Hierzu antworteten 72,3 % (*n* = 34) der TN. Am Häufigsten wurde die Berufserfahrung (55,9 %, *n* = 19) im Fachbereich genannt, die teilweise mit > 1 bis ≥ 5 Jahre beschrieben wurde. Ergänzend wurden u. a. Fachkenntnisse, eine abgeschlossene FWB, Kenntnisse in der Pflegeplanung sowie eine pädagogische oder pflegewissenschaftliche Qualifikation aufgelistet.

### Beratung und Begleitung in der PV in t_1_ und t_0_

Für TN, die nicht aktiv an einer PV teilnahmen, endete die Befragung an dieser Stelle.

Die Frage, ob es von den visitierenden Personen ein Schulungsangebot oder spezielle Hinweise zu innerbetrieblichen Fortbildungen gab, wurde in t_1_ von 4,4 % (*n* = 2) und in t_0_ von 43,3 % (*n* = 13) der TN bejaht *(ESM 2)*. Ein spezifisches Feedback im Rahmen der PV erhielten in t_1_ 53,3 % (*n* = 24) und in t_0_ 70,0 % (*n* = 21) der TN *(ESM 2).* Diejenigen TN, die ein Feedback erhielten, gaben zu 75,0 % (*n* = 18; t_1_) und zu 100 % (*n* = 21; t_0_) an, dass ihnen dies für ihr weiteres pflegerisches Handeln half *(ESM 2)*. Einen positiven Einfluss der PV auf die Übergabe gaben in t_1_ 48,9 % (*n* = 22) und in t_0_ 53,3 % (*n* = 16) der TN an *(ESM 2).*

### Gesamtbewertung der PV

**Abb. 6:**
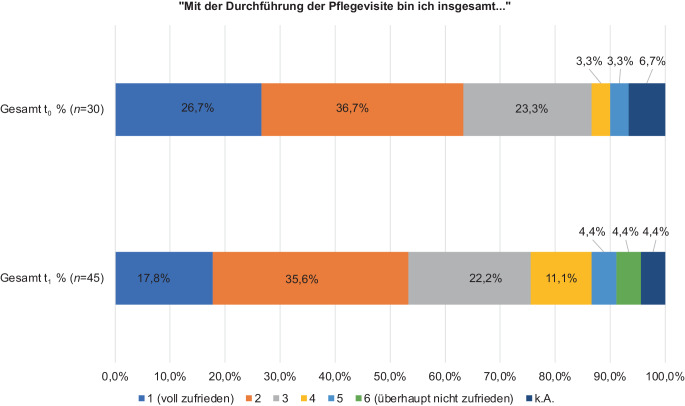
Bewertung der Pflegevisite von Pflegenden nach (t_1_) und vor Implementierung (t_0_) von Prozessverantwortlicher Pflege

Die PV wurde in t_1_ und t_0_ im Median mit einer 2 bewertet (IQR t_1_: 1–3; t_0_: 1–2) und im Mittelwert in t_1_ mit 2,60 (SD: 1,31) sowie in t_0_ mit 2,14 (SD: 1,07; Abb. 6)*.*

Insgesamt 21,3 % (*n* = 10) der TN gaben in t_1_ eine schriftliche Rückmeldung zu Verbesserungsvorschlägen im Rahmen der PV. Dabei war der häufigste Wunsch, die Kontinuität des wöchentlichen Rhythmus (40 %, *n* = 4) einzuhalten. Außerdem sollte die PV noch besser auf den einzelnen Patientinnen- und Patientenfall abgestimmt werden.

### Anzahl und Dauer der PV

Im Jahr 2023 wurde bei insgesamt 254 Patientinnen und Patienten eine PV durchgeführt, von denen 70,5 % (*n* = 179) in PP-Betreuung waren. In 53,9 % der Fälle (*n* = 137) gab es eine oder mehrere Revisiten im Verlauf des Aufenthalts auf der ITS. Die PV dauerte bei 99,2 % (*n* = 252) der Patientinnen und Patienten ≤ 30 Min und bei 0,8 % (*n* = 2) der Patientinnen und Patienten > 30 Min.

## Diskussion

Diese Evaluationsstudie hatte zum Ziel, die Auswirkungen der PV auf die PP aus Sicht der Pflegenden zu untersuchen. Als sekundäre Ziele sollten u. a. die weiteren Auswirkungen der PV und eine Gesamtbewertung mit einem Vergleich zu den Ergebnissen der Pilotierungsphase ermittelt werden. Im Ergebnis zeigte sich, dass die PV dazu beiträgt, die Pflegeplanung bei prozessverantwortlich betreuten Patientinnen und Patienten zu evaluieren und dass sie eine Unterstützung für die Umsetzung von PP darstellt. Die Auswirkungen der PV sowie die Gesamtbewertung sind positiv und ähneln sich in t_1_ und t_0_, sodass kein statistisch signifikanter Unterschied festgestellt werden konnte.

Insgesamt nahmen an der Evaluationsstudie Pflegende teil, die die Grundgesamtheit der Pilot-ITS abbilden. Die PV erleichtert und ermöglicht den PP, ihre Aufgabe bzw. Rolle, das Konzept PP sowie die Pflegeplanung besser wahrzunehmen, was zum Teil auch im Kontext der Langzeitversorgung [25] beschrieben wird. Die qualitätserhaltende bzw. -verbessernde Funktion der PV [2] könnte darauf ebenfalls einen Einfluss haben. Da die Anzahl der teilnehmenden PP im Gegensatz zu P gering war, müssen auch statistisch nichtsignifikante Unterschiede der beiden Gruppen grundsätzlich mit Vorsicht interpretiert werden. Losgelöst davon gaben alle teilnehmenden PP an, dass die PV dazu beiträgt, die Pflegeplanung bei prozessverantwortlich betreuten Patientinnen und Patienten zu evaluieren, und eine Unterstützung für die Umsetzung von PP darstellt.

Eine mögliche Verbesserung der Pflegeplanung durch die PV wurde heterogen beantwortet. Möglicherweise hatte die intensive Informationspolitik, Unterstützung und Schulung der PP im Rahmen der Entwicklung und Implementierung [20] hier einen Einfluss. Parallel beschreiben Zurbrügg und Lüdi-Conti [26] Synergieeffekte von PV und PP, da die PP eine individuelle Pflegeplanung erstellt und die PV ggf. Optimierungspotenziale aufzeigt.

Der positive Einfluss der PV auf die Patientinnen- und Patientenzentrierung ist vergleichbar mit den Erkenntnissen von Görres et al. [2], allerdings lässt sich zwischen t_1_ und t_0_ ein leichter Zustimmungsrückgang von rund 10 % verzeichnen, der statistisch nicht signifikant ist. Das könnte auf das standardisierte Vorgehen in der PV zurückgeführt werden. Die Durchführung der PV sollte somit mehr individualisiert und deren Auswirkung auf die Patientinnen- und Patientenzentrierung auch in zukünftigen Studien als Outcomeparameter adressiert werden. Die bewusstere Anpassung der Pflegemaßnahmen an die Bedürfnisse der Patientinnen und Patienten zeigen hingegen ähnliche Ergebnisse. Dies könnte im PP-Konzept begründet sein, da die PP sensitiver für Adaptionen im Pflegeplan ist.

Die Verbesserung der Pflegequalität durch die PV, die sich vor allem in der Patientinnen- und Patientenorientierung zeigt, wurde in t_1_ und t_0_ mit ähnlicher Zustimmung abgebildet. Dies kann durch die Überprüfungsfunktion der PV in Belangen der pflegerischen Leistung und somit der Pflegequalität herbeigeführt worden sein [2, 3]. Hervorzuheben ist der statistisch signifikante Effekt der PV auf die Verbesserung der Pflegequalität und Pflegeplanung bei prozessverantwortlich betreuten Patientinnen und Patienten. Dieses Ergebnis darf, wenn auch mit Vorsicht, positiv und als erster Hinweis für einen Effekt der PV auf die PP gewertet werden. Hier könnten Langzeitstudien hilfreich sein, um nachhaltige Auswirkungen der PV auf die Pflegequalität anhand noch festzulegender pflegesensitiver Outcomes zu beurteilen.

Der wöchentliche Rhythmus mit einem Zeitrahmen von rund 30 Min deckt sich annähernd mit den weiteren Erkenntnissen [2, 13, 17], Empfehlungen [27] und gewonnenen Daten aus der Umsetzung der PV im Jahr 2023. Abweichend davon ist die Angabe zur Anzahl der zusätzlich zum Einsatz kommenden Personen bei der PV, die auch international unterschiedlich angegeben wird [7, 9, 11]. Eine Tendenz hin zu einer visitierenden Person gab es bereits in t_0_, jedoch fand die PV hier immer mit 2 Personen statt [19] Die Reduzierung von Dauer und Anzahl der visitierenden Personen könnte jedoch auch auf eine effizientere Durchführung der PV hindeuten.

Die Einschlusskriterien für eine PV sind in t_1_ und t_0_ ähnlich und sollten daher als Voraussetzung bestehen bleiben. Das Team der visitierenden Personen spiegelt sich in den Angaben der TN zu den Qualifikationen gut wider. Eine langjährige Berufserfahrung und der Erwerb von weiterführenden Qualifikationen sind auch im Kontext der Übernahme der Versorgung von hochkomplexen Versorgungssituationen zu finden [28, 29].

Durch die PV können positive Auswirkungen auf die Nutzung von Fort- und Weiterbildungsmöglichkeiten entstehen [8, 9]. Während in t_1_ die meisten TN dies verneinten, bejaht in t_0_ noch rund die Hälfte der Befragten, dass sie einen Hinweis auf Fortbildungen erhielten. Hier sollte nachgesteuert werden, um einem möglichen Wissensverlust [8] beim zwingend notwendigen Aufbau von Handlungskompetenzen in der Versorgung kritischen kranker Patientinnen und Patienten [10] entgegenzuwirken. Hierzu wurden die visitierenden Personen sensibilisiert. Überdies sollen die Ergebnisse dieser Studie in klinikinternen Fortbildungen vorgestellt und diskutiert werden. Die persönliche Entwicklung der Pflegenden kann durch ein spezifisches Feedback [28] der visitierenden Personen begünstigt werden [25]. An dieser Stelle ist ein größeres Entfaltungspotenzial vorhanden.

Die Gesamtbewertung der PV fiel in t_1_ etwas schlechter aus als in t_0_, kann jedoch weiterhin positiv gewertet werden. Es sollte zukünftig eine Ausfallstrategie im Dienstplan berücksichtigt werden, die die wöchentliche PV sicherstellt und z. B. krankheitsbedingte Ausfälle kompensiert.

### Stärken und Limitationen

Die Ergebnisse dieser Evaluationsstudie geben einen ersten wichtigen Hinweis zu den Auswirkungen der PV auf die PP im Setting der ITS. Ein Großteil der TN hat darüber hinaus die PV in der Praxis erlebt und der Rücklauf ist positiv zu bewerten.

Gleichwohl lassen sich auch Limitationen beschreiben. Der eingesetzte Fragebogen war nicht validiert, wurde jedoch einem standardisierten Pretest unterzogen. Dies kann einen Einfluss auf die generierten Ergebnisse haben. In t_1_ waren von 47 TN nur 8 PP. Hervorzuheben ist jedoch, dass lediglich 20 % (*n* = 2) der PP nicht an dieser Studie teilnahmen. Eine größere Anzahl von PP könnte die Ergebnisse aussagekräftiger machen. Im Zeitraum der Datenaufnahme konnten abwesenheitsbedingt nicht alle Pflegenden der Pilot-ITS eingeschlossen werden. In t_0_ fand im Vergleich zu t_1_ die PV immer mit 2 Personen und die Dokumentation nicht im PDMS, sondern händisch im *Pen-and-paper*-Format statt.

## Fazit für die Praxis

- Die PV kann bei der Umsetzung von PP auf der ITS sowie der Evaluation der Pflegeplanung unterstützen.
- Die PV kann einen positiven Einfluss auf die Pflegequalität haben.
- Die PV sollte wöchentlich durch Pflegende mit umfassender Expertise im Fachbereich der ITS stattfinden.
- Die PV sollte generell individualisiert auf die Situation der Patientinnen und Patienten ausgerichtet werden.

## Supplementary Information

ESM_1_: Qualifikation und Berufserfahrung der teilnehmenden Pflegenden in t_1_, ESM_2_: Tabellen mit detaillierten Ergebnissen zu weiteren Auswirkungen und Rahmenbedingungen der Pflegevisite

## Data Availability

Alle in dieser Studie generierten Daten werden innerhalb der Veröffentlichung zur Verfügung gestellt.

## Abkürzungen

HDZ NRW: Herz- und Diabeteszentrum NRW, Universitätsklinikum der Ruhr-Universität Bochum
ITS: Intensivstation
IQR: Interquartilsabstand
P: Pflegende ohne Prozessverantwortung
PDMS: Patient-data-management-System
PN: Primary Nursing
PP: Prozessverantwortliche Pflege/prozessverantwortlich Pflegende
PV: Pflegevisite
SD: Standardabweichung
TN: Teilnehmende
